# Sacrificial-template-free synthesis of core-shell C@Bi_2_S_3_ heterostructures for efficient supercapacitor and H_2_ production applications

**DOI:** 10.1038/s41598-018-22622-0

**Published:** 2018-03-08

**Authors:** S. V. Prabhakar Vattikuti, Anil Kumar Reddy Police, Jaesool Shim, Chan Byon

**Affiliations:** 1 School of Mechanical Engineering, Yeungnam University, Gyeongsan, 712-749 South Korea; 2 School of Mechanical and Nuclear Engineering, Ulsan National Institute of Science and Technology (UNIST), Ulsan, 44919 Republic of Korea

## Abstract

Core-shell heterostructures have attracted considerable attention owing to their unique properties and broad range of applications in lithium ion batteries, supercapacitors, and catalysis. Conversely, the effective synthesis of Bi_2_S_3_ nanorod core@ amorphous carbon shell heterostructure remains an important challenge. In this study, C@Bi_2_S_3_ core-shell heterostructures with enhanced supercapacitor performance were synthesized via sacrificial- template-free one-pot-synthesis method. The highest specific capacities of the C@Bi_2_S_3_ core shell was 333.43 F g^−1^ at a current density of 1 A g^−1^. Core-shell-structured C@Bi_2_S_3_ exhibits 1.86 times higher photocatalytic H_2_ production than the pristine Bi_2_S_3_ under simulated solar light irradiation. This core-shell feature of C@Bi_2_S_3_ provides efficient charge separation and transfer owing to the formed heterojunction and a short radial transfer path, thus efficiently diminishing the charge recombination; it also facilitates plenty of active sites for the hydrogen evolution reaction owing to its mesoporous nature. These outcomes will open opportunities for developing low-cost and noble-metal-free efficient electrode materials for water splitting and supercapacitor applications.

## Introduction

Active supercapacitors are a class of energy storage devices that have fascinated the scientific community owing to their high power capacity with long cycle life, low cost, and low maintenance. Recently, supercapacitors are being widely used in memory backup, consumer electronics, and industrial power management^1–3^. In addition, supercapacitors are safer and have a lesser environmental impact than batteries^2,3^. Mainly, the specific capacitance of a supercapacitor depends on the electrode materials and thus, electrode materials of supercapacitors play an important role. There are several electrode materials available for supercapacitors; the most common are carbon materials owing to their high conductivity, large specific surface area, different forms, and abundance. However, their activity is not sufficient, owing to their low specific energy. In addition, the inset surface of carbon materials makes it difficult for electrolytes to further penetrate the internal layers of carbon^4^. Different approaches have been employed to overcome these issues like introduction of metal oxides, hetero atoms, and coupling of two or more materials.

In recent years, heterostructure nanomaterials comprising two or three different functionalities showed enhanced or different physicochemical performances compared to single functionality materials^5^. Among the various heterostructure features, core-shell structures exhibit superior performances owing to the combined effects of cores and shells^6,7^. Meanwhile, metal sulfides including nickel sulfides, cobalt sulfides, and bismuth disulfides are very important semiconductor materials and have been employed to develop supercapacitor electrodes^8–11^. Among these, bismuth sulfide (Bi_2_S_3_) has a lamellar structure with a 1.3–1.7 eV direct band gap and these features offers potential applications in the field of thermoelectric cooler devices^12^, lithium-ion batteries^13^, and optoelectronic devices^14^ owing to the possibility of band gap tuning with different sizes of the subcomponent. As the above-mentioned properties strongly rely on the size, shape and morphologies, diverse strategies are put forth to controlled synthesis of bismuth sulfide nanostructured materials. Several Bi_2_S_3_ nanomaterials like nano rods, tubes, wires, ribbons are prepared by various synthesis procedures. However, Bi_2_S_3_ as an electrode material possesses low conductivity as well as high volume expansion during the charge and discharge cycling, which are major challenges. In addition, the reversible specific capacity fades rapidly during cycling reactions in metal sulfides. This capacitance loss is mainly because of the volume expansion during the charge and discharge processes causing particle cracks and pulverization that breakdown the electrical contacts in the bismuth sulfide anode. Hence, improving the number of charge discharge cycles is a crucial task to address for the optimization of Bi_2_S_3_ based anode materials for supercapacitor applications.

To overcome these drawbacks, different methods have been followed to enhance its electrochemical activity; one of the most efficient ways is to design nanohybrids with a core-shell structure using the allotropes of carbon materials, which can act as a projective/buffer layer to improve the volume expansion during charge and discharge processes^15,16^. Recently, Yang *et al*.^17^ fabricated strongly coupled Bi_2_S_3_ nanocrystals anchored on carbon nanotube (CNT) backbones; the resulting Bi_2_S_3_@CNT enhanced the conductivity. Zhang *et al*.^18^ fabricated composites with CNTs twined with Bi_2_S_3_ microspheres using a facile reflux synthetic route, and the CNTs@Bi_2_S_3_ composite electrode showed a reversible specific capacity of 247.9 mA h g^−1^ after 50 cycles. Jung *et al*.^19^ and Jin *et al*.^20^ developed carbon-coated Bi_2_S_3_ crystallites as electrode materials; the specific capacity and cyclability of C@Bi_2_S_3_ hybrid were significantly enhanced compared to pristine Bi_2_S_3_. However, many well-known electrode materials with unique structural integrity for supercapacitor applications remain unexplored to enhance the specific capacitance. Continuing with the development of carbon nanomaterials, graphene and CNTs are often referred to as supercapacitors owing to their high electron-storage capacities and good electrical conductivity^21,22^.

Visible-light-driven photocatalytic reforming of biomass-derived feedstock is a sustainable, renewable, and cost-effective method for H_2_ production. Recently, the enhanced photocatalytic activity of Bi_2_S_3_-based composites were explored for photocatalytic hydrogen production^23–25^. Kadam *et al*.^26^ reported a Bi_2_S_3_ quantum dot/glass composite that demonstrated efficient hydrogen production (6418.8 μmol h^−1^ g^−1^) under solar light irradiation. Feng *et al*.^27^ reported carbon-coated Co_9_S_8_ nanoparticles as electrocatalysts for water splitting with trithiocyanuric acid as a surfactant for the formation of core-shell structures. However, surfactant- or sacrificial- template-free synthetic procedures remains a challenge, offering scope for the rational design of high-performance core-shell heterostructures.

In this work, we have attempted and reported a rational assembly of carbon on the surface of Bi_2_S_3_ nanorods, formed as a core-shell heterostructure via a facile and simple one-step hydrothermal reaction. The resulting C@Bi_2_S_3_ core-shell heterostructures contain well-defined cross linkages between the Bi_2_S_3_ core and carbon shell. These highly porous structures allow the free permeation of electrolyte ensuring rapid movement of ions. Also, the carbon shell on the Bi_2_S_3_ core acts an additive effectually shielding the mechanical strain during the charge discharge processes. Moreover, the crosslinked assemblies in the C@Bi_2_S_3_ core-shell heterostructures facilitates charge transfer through multipath electron transfer, which could accelerate the electrochemical processes to enhance high energy storage and photocatalytic H_2_ evolution features.

## Experimental

### **Synthesis of Bi**_**2**_**S**_**3**_**and C@Bi**_**2**_**S**_**3**_

To develop the final C@Bi_2_S_3_, the Bi_2_S_3_ was initially synthesized as described below using a modified hydrothermal method from previously reported literature^28^. In detail, Bi(NO_3_)_3_·5H_2_O (0.2 M) is dissolved in 25 mL of deionized (DI) water in a glass beaker with vigorous stirring, and a mixture of sodium sulfide (Na_2_S·9H_2_O) (0.3 M) and 36 mL H_2_O_2_ was added drop by drop into the above solution at 90 °C with continuous stirring for 1 h. Then, it is transferred to a Teflon-lined autoclave and maintained at 190 °C for 6 h. Once it is cooled to room temperature naturally, the product was collected by centrifuging at 6000 rpm, and then was washed three times with ethanol. Then, the product was dried in a vacuum oven at 100 °C for 3 h.

**Figure 1:**
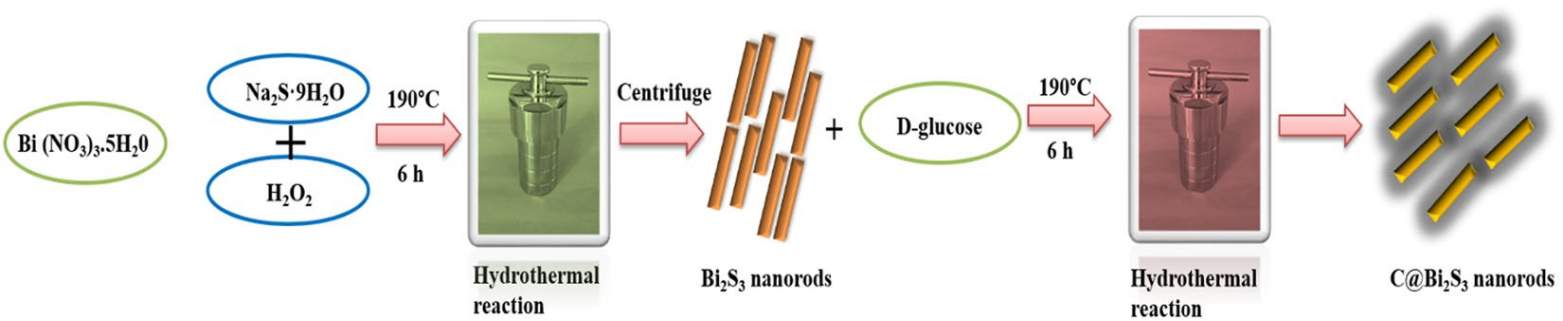
Schematic synthetic procedure for formation of C@Bi_2_S_3_ core-shell structure.

In a typical synthetic procedure for C@Bi_2_S_3_, 5 mg of the as-prepared Bi_2_S_3_ was added in 30 mL ethanol and stirred to ensure homogenous mixing. Then, 15 mL of D-glucose solution (0.02 M) was mixed with the above solution, followed by continuous stirring for 60 min, and then transferred into a Teflon-lined stainless-steel autoclave and maintained at 190 °C for 6 h, and then cooled to room temperature naturally. The effect of hold time was explored by interrupting the shell growth at different times: 4, 6, 8, and 12 h, with the other parameters kept constant. Finally, the products were washed thoroughly with ethanol and water thrice and dried at 100 °C for 3 h. The schematic of the preparation procedures for the C@Bi_2_S_3_ is presented in Fig. 1.

### Characterization

The as-synthesized samples were characterized by X-ray powder diffraction (XRD) using a Shimadzu Labx X-ray diffractometer, XRD 6100 model. The XRD patterns of the samples were obtained using Cu Kα radiation (λ = 0.154 nm) at 40 kV and 30 mA. Transmission electron microscopy (TEM) and high-resolution transmission electron microscopy (HRTEM) observations were performed with a Hitachi H-7000 at 110 kV and a Tecnai G2 F 20 s-twin TEM at an accelerating voltage of 300 kV, respectively. Energy-dispersive X-ray spectroscopy (EDS) was performed to analyze the elemental composition. The scanning electron microscope (SEM) images were obtained using a Hitachi S-4800. X-ray photoelectron spectroscopy (XPS) analysis was conducted using a Thermo Scientific instrument and K-alpha surface analysis. The nitrogen adsorption–desorption isotherms were recorded using a Micromeritics ASAP 2420 surface area analyzer at liquid nitrogen temperature. Prior to gas adsorption, all photocatalysts were degassed for 3 h at 200 °C. The infrared (IR) spectra were recorded using an Avatar 370 Fourier transform infrared (FTIR) spectroscope. The spectra were acquired at a resolution of 4 cm^−1^ in the spectral range of 4000–400 cm^−1^ using 32 scans. Raman spectra were verified using a Jobin-Yvon Horiba (Lab Ram HR-800) single monochromator attached with a Peltier-cooled charged coupled device (CCD) and used 532 nm Argon (Ar^+^) laser excitation source at 25 °C under back scattering geometry.

### 2.3 Electrochemical Performance Measurements

All the electrochemical measurements were performed using the electrochemical workstation (Biologic SP-200) using a three-electrode configuration in a 0.5 M Na_2_SO_4_ aqueous electrolyte. The as-synthesized samples supported on ITO substrate directly served as the working electrode, while a saturated calomel electrode (SCE) and a platinum wire were used as the reference and the counter electrodes, respectively. Cyclic voltammetry (CV) and galvanostatic charge−discharge (GCD) measurements were performed to examine the electrochemical performance of the working electrodes. Electrochemical impedance spectroscopy (EIS) was conducted by applying an alternating current voltage with a 0-mV amplitude in the frequency range of 0.01 Hz to 1 MHz. The specific capacities (Cs, C g^−1^) of the Bi_2_S_3_ samples were estimated from the GCD curves based on the following equation:

1 $${C}_{s}=\frac{{\rm{I}}\,\times \,{\rm{\Delta }}{\rm{t}}}{{\rm{m}}},$$

where I (mA) indicates the discharge current, Δt (s) refers to the discharge time, and m (mg) corresponds to the mass of the active material. The corresponding specific capacitance can be estimated according to the following equation:

2 $$C=\frac{2{\rm{I}}}{{{\rm{m}}{\rm{\Delta }}{\rm{V}}}^{2}}{\int }_{{t}_{1}}^{{t}_{2}}{\rm{V}}(t)dt,$$

where ΔV (V) is the potential range, V(t) is the operating of potential, and t_1_ and t_2_ denote to the initial and terminational discharge time of the GCD curves, respectively.

### **Photocatalytic H**_**2**_**production**

Using the as-prepared photocatalysts, the photocatalytic hydrogen production was carried out by water splitting under simulated solar light irradiation. The reaction was carried out in a 150-mL tube-like quartz reactor with a round bottom. 5 mg of the photocatalyst was added to 50 mL of 5% aqueous lactic acid solution in a quartz reactor, which was then sealed with an air-tight rubber septum. The reactor was evacuated for 20 min, and the solution was purged with N_2_ gas for 20 min. The reaction was carried out using a 300 W Xe lamp (Max 303) with a light intensity of 50 mW cm^−2^. The amount of H_2_ gas generated during the reaction was monitored at one-hour intervals by collecting the gas samples in an airtight syringe. The analysis of the sample was carried out using a gas chromatograph (6500GC system-YL Instrument) equipped with a thermal conductivity (TC) detector and a molecular sieve 5A column with He as the carrier gas.

## Results and Discussion

On the basis of the attempted experimental analysis, the formation mechanism of the core-shell structure and accompanying morphology evolution process is illustrated in Fig. 1. In this work, D-glucose was selected as a carbon source owing to its (i) environmental biocompatibility, (ii) unique and stable polymerized structure, and (iii) abundance. Moreover, the *in-situ* carbon coating also considerably simplifies the process and reduces the cost. Generally, D-glucose serving as a surfactant can generate carbon via a solid thermal decomposition reaction^29^. In the present synthesis methodology, we used ethanol as a solvent to disperse the Bi_2_S_3_ nanorods and D-glucose was introduced as the carbon source. When the D-glucose is subjected to the hydrothermal treatment at 190 °C, more than the glycosidation temperature, it tends to form C-C bonding that leads to the aromatization and carbonation. Initially, the glucose molecules undergo polymerization to form aromatic compounds or oligosaccharides. As the solution reach critical supersaturation, crosslinking between these macromolecules tends to the carbonization that results in a short single burst of nucleation. Here, the highly dispersed Bi_2_S_3_ nanorods, possessing highly reactive surface exposed outside, offers its surface to catalyze the carbonization of glucose (nucleation) that leads to *in-situ* deposition of carbon products around the Bi_2_S_3_ nanorods to produce carbonaceous shell. The growth of nuclei is subject to the reaction temperature, time and concentration. Herein, we studied the effect of reaction time (4, 6, 8, and 12 h holding times), keeping the other two conditions constant, on the thickness of the carbon shell and discussed in the HRTEM section. Under the complex polymerization of D-glucose at hydrothermal conditions (4, 6, 8, and 12 h holding times), Bi_2_S_3_ nanorod arrays employed as the templates form scalable core-shell C@ Bi_2_S_3_, where Bi_2_S_3_ nanorods are initially coated by carbonaceous nucleation on their outer surface. Under hydrothermal treatment, the D-glucose was degraded to carbon and anchored onto the surface of Bi_2_S_3_ nanorods^13^. As reported in the literature, the thickness of the carbonaceous layer typically depends upon the synthesis conditions^30^. For example, a glucose-derived carbon precursor can be readily integrated onto nanostructures in solution, which is carbothermally reduced to metallic ions by carbon with respect to hydrothermal temperature^30^. Saravanakumar *et al*.^29^ demonstrated dextrose as a carbon source for the synthesis of carbon-coated V_2_O_5_ nanorods. They demonstrated that the controlled hydrothermal treatment led the formation of rod-like carbon structures. In this work, the overall carbonization process is controlled based on the holding time to avoid the destruction of Bi_2_S_3_ nanorod arrays. It is well known that above 220 °C, sulfur is decomposed and oxides were formed. Therefore, we fixed the hydrothermal temperature at 190 °C and studied the effect of holding time on the carbonaceous layer formation.

**Figure 2:**
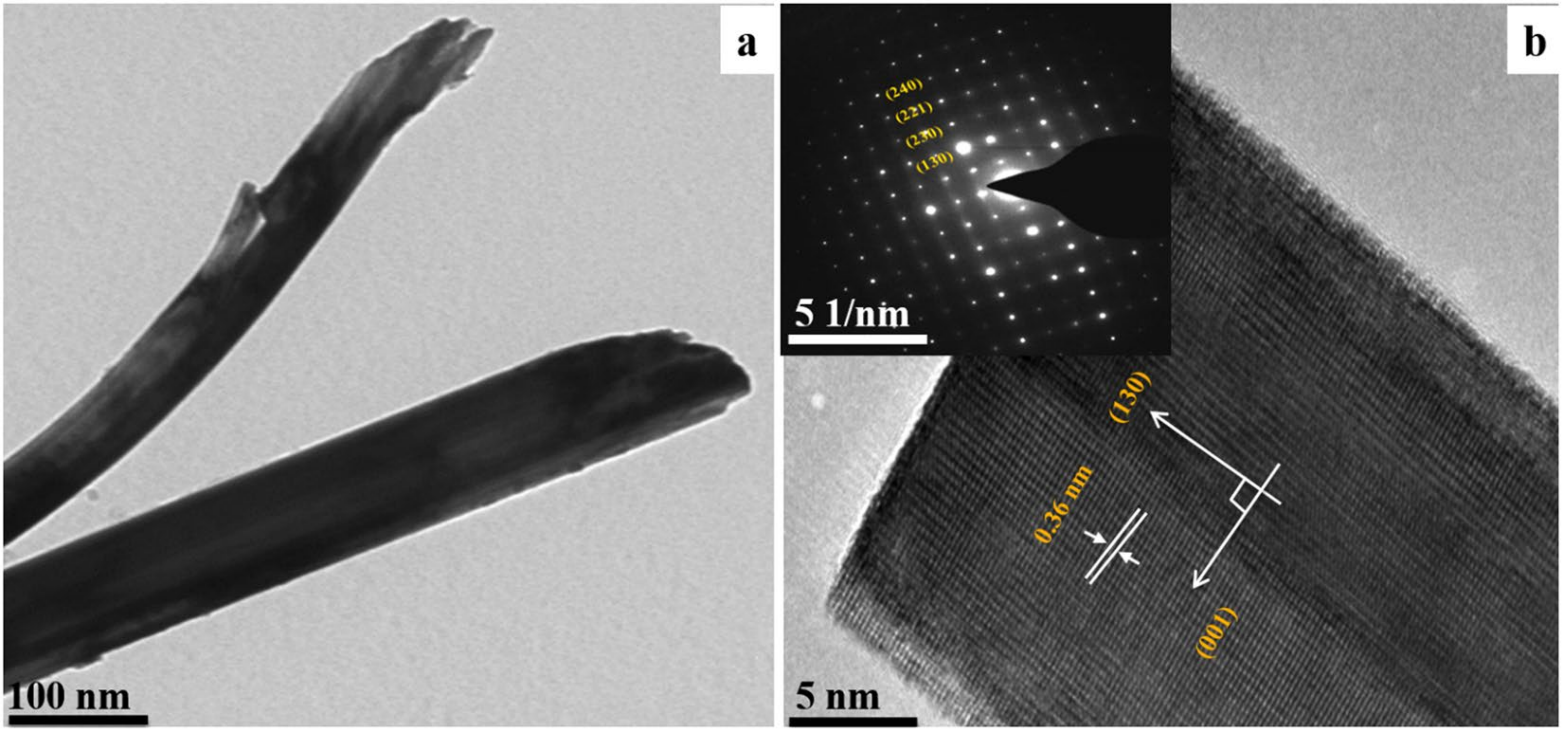
HRTEM image of the pristine Bi_2_S_3_ nanorods.

The morphological studies were performed using HRTEM and SEM. A representative HRTEM image of pristine Bi_2_S_3_ is observed in Fig. 2. As can be seen, the nanorods are a few nanometers in length (Fig. 2a). The high-magnification HRTEM image (Fig. 2b) indicates high crystallinity of the Bi_2_S_3_ nanorods with a lattice spacing of 0.36 nm that can be assigned to the (130) plane of orthorhombic Bi_2_S_3_, indicating that Bi_2_S_3_ nanorods favorably grew parallel direction to the (130) plane. The inset in Fig. 2b presents the relative SAED image reveals some bright spot patterns of pristine Bi_2_S_3_, which is also consistent with the single crystalline Bi_2_S_3_ indexed as the (130) zone c-axis^28^.

**Figure 3:**
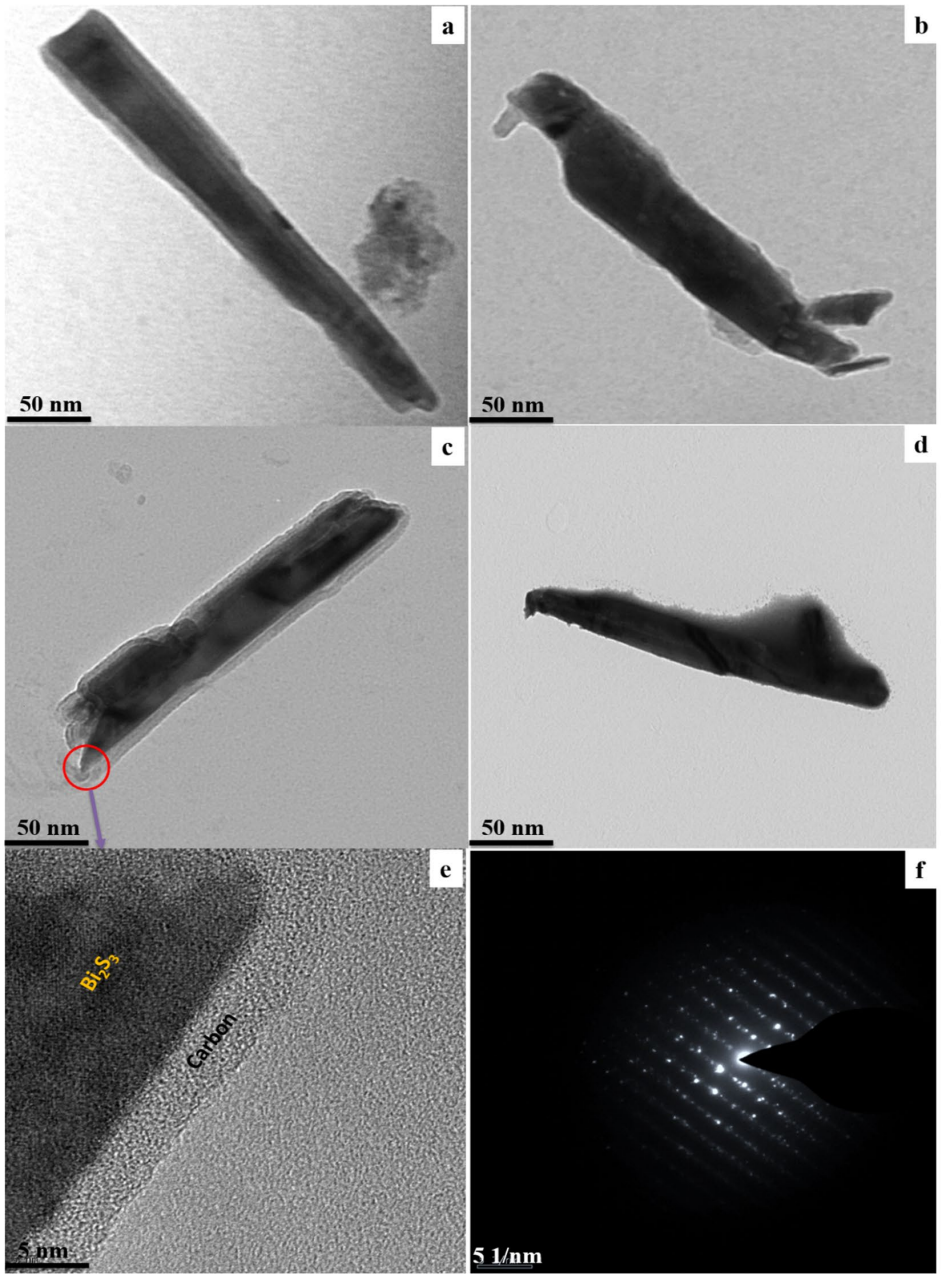
HRTEM image of the growth of C@Bi_2_S_3_ at different holding times (**a**) 4, (**b**) 6, (**c**) 8, and (**d**) 12 h, (**e**) high magnification image and (**f**) SAED pattern of C@Bi_2_S_3_ at a holding time of 8 h.

**Figure 4:**
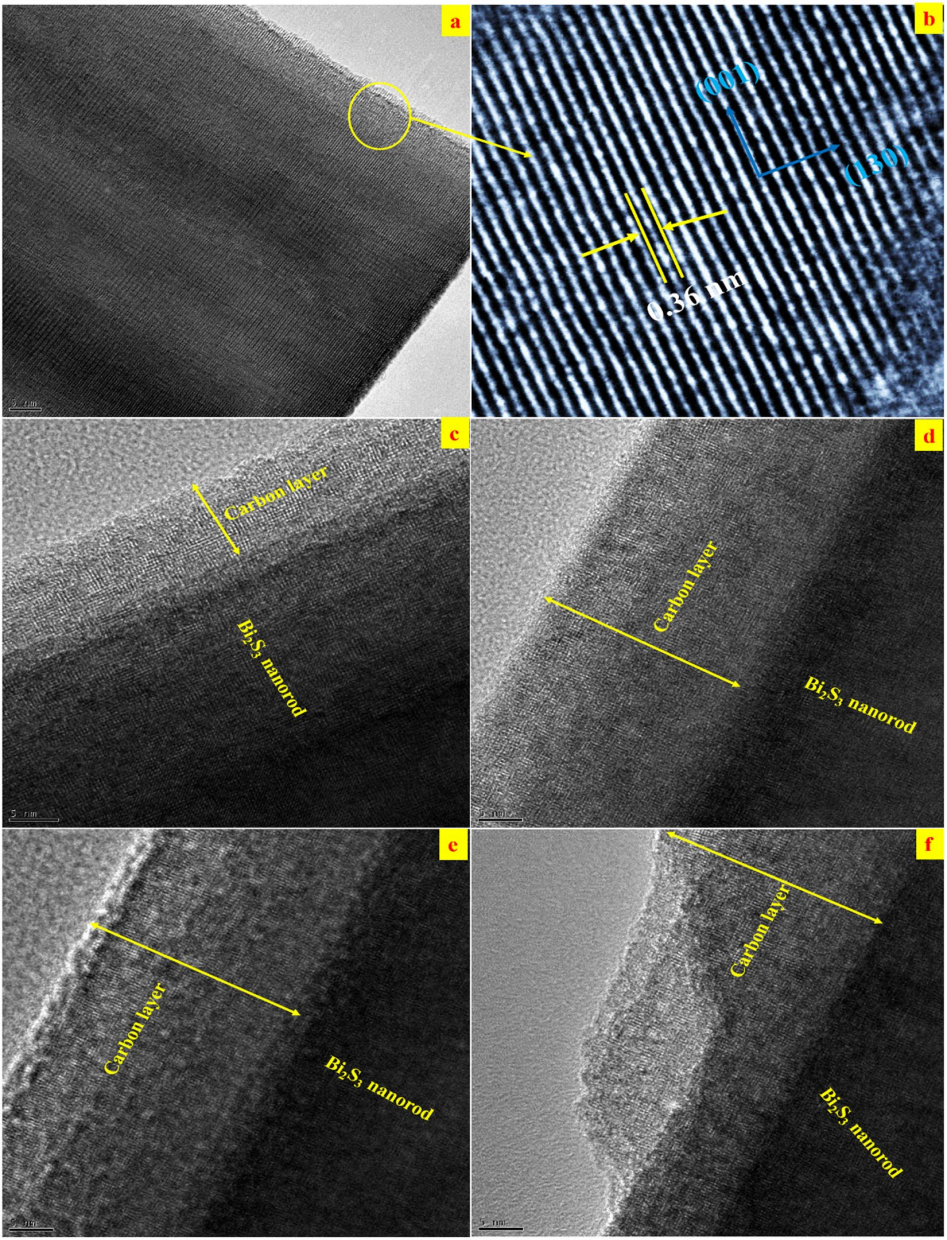
High magnification HRTEM image of the (**a,b**) pristine Bi_2_S_3_ and C@Bi_2_S_3_ at holding times of (**c**) 4 h, (**d**) 6 h, (**e**) 8 h and (**f**) 12 h.

The decoration of the carbon layer on the surface of individual Bi_2_S_3_ nanorods with respect to the holding time is examined via HRTEM. Figure 3 shows the HRTEM images of C@Bi_2_S_3_ samples. The nanorods are fully enclosed by the carbon material, thus forming a core-shell structure. In the case of the C@Bi_2_S_3_ sample, a typical core-shell shape of organic/inorganic hybrid nanostructure is formed, with well-organized atomic structures of carbon layers on the surface of Bi_2_S_3_ nanorod. It can be inferred that the ultra-thin shell-like carbon layer is formed by the nucleation of carbonaceous molecules. Figure 4 shows the high-magnification HRTEM images of pristine Bi_2_S_3_ and C@Bi_2_S_3_ at different holding times: 4, 6, 8, and 12 h. It is noteworthy that as the holding time is increased, the thickness of the carbon layer increases. Moreover, on the Bi_2_S_3_ nanorod surface, there is a ~2 nm thick coating layer, which is probably amorphous carbon resulting from the polymerization of glucose is seen for all the C@Bi_2_S_3_ samples. It was observed that the thickness of the carbon layer slightly increased with an increase in the holding time. However, the carbon layer becomes dense with increasing holding times which is due to the excess carbon particles deposited on the Bi_2_S_3_ nanorod. The HRTEM images depict a dense carbon layer on the Bi_2_S_3_ nanorods surface as highlighted by the yellow arrows. HR-TEM performed on the grown carbon layer on the Bi_2_S_3_ nanorods surface reveals its structural uniformity and amorphous nature. At different holding times, the Bi_2_S_3_ rods were assembled by the amorphous carbon particles with variable thickness and densities. In addition, the mesoporous features of the carbon layer attributed roughness to the surfaces of the Bi_2_S_3_ nanorods, leading to enhanced electrochemical properties. In other words, the homogeneous carbon layer structure could efficiently enhance the conductivity of the electrodes, limit the volume change of Bi_2_S_3_ materials, and keep the Bi_2_S_3_ electrode stable during oxidation–reduction reactions. Based on the HRTEM analysis, the C@Bi_2_S_3_ sample at an 8 h holding time is considered for further characterization and applications in this work.

**Figure 5:**
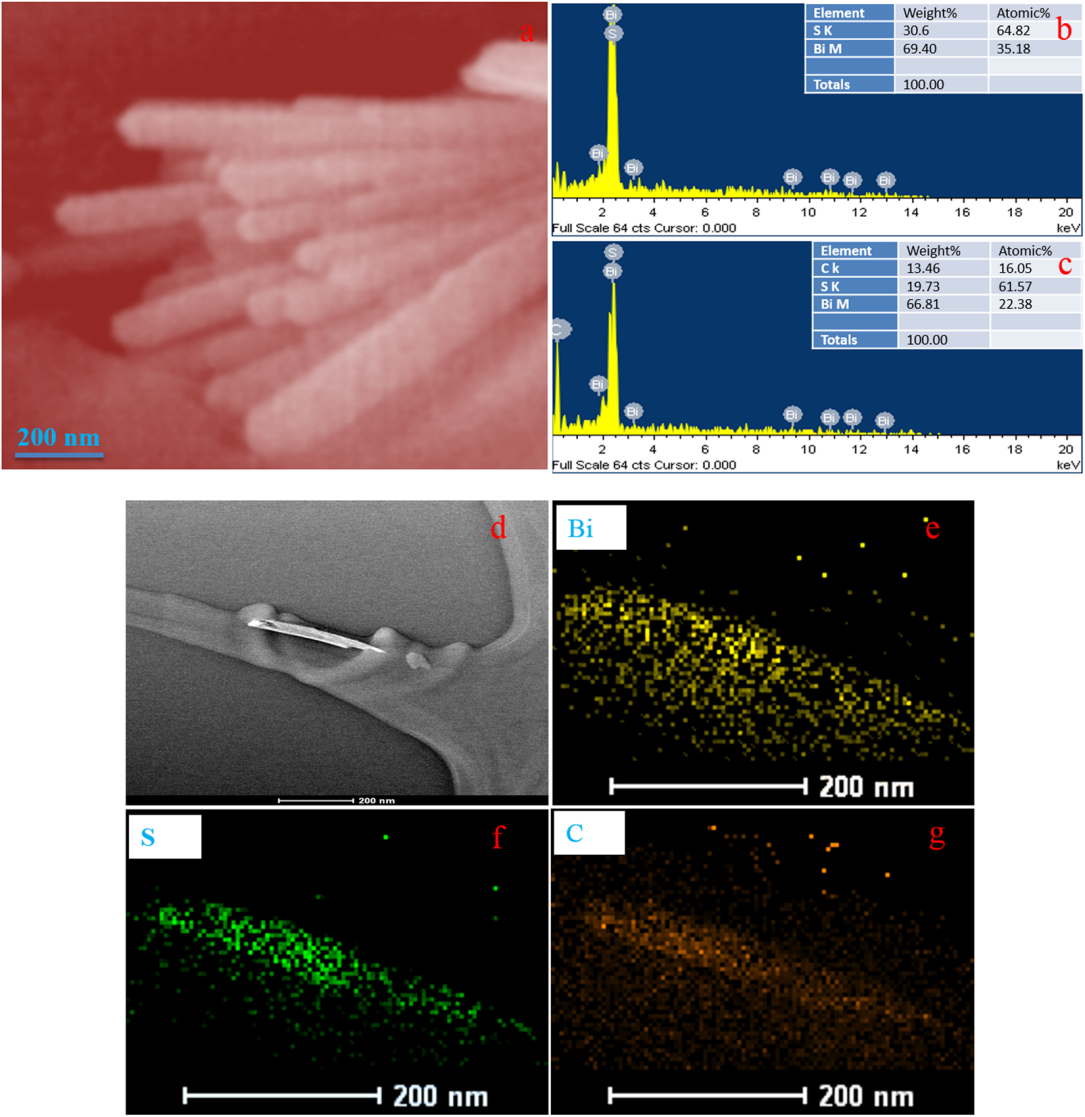
(**a**) SEM image of C@Bi_2_S_3_ holding time at 8 h, (**b,c**) EDX mapping of the pristine Bi_2_S_3_ and C@Bi_2_S_3_ at 8 h, (**d–g**) HRTEM mapping images.

Figure 5a shows the SEM images of C@Bi_2_S_3_ sample. The SEM images indicate that the C@Bi_2_S_3_ structure comprises a large amount of homogeneous nanorods, which are covered by the amorphous carbon material. SEM observation indicates that the carbon was densely packed and uniformly covered the entire surface of Bi_2_S_3_ nanorods, resulting in the formation of core-shell structures. SEM images show that the nanorods have a length of approximately a few nanometers to submicron. Figure 5(b,c) presents the EDX elemental mapping of pristine Bi_2_S_3_ and C@Bi_2_S_3_ samples. This result also confirmed the deposition of carbon materials throughout the Bi_2_S_3_ nanorods and provides evidence for the existence of carbon materials in the C@Bi_2_S_3_ sample. Furthermore, the HRTEM elemental mapping of the C@Bi_2_S_3_ sample is shown in Fig. 5(d–g). It can be seen that the Bi_2_S_3_ nanorod is also decorated with the carbon layer.

**Figure 6:**
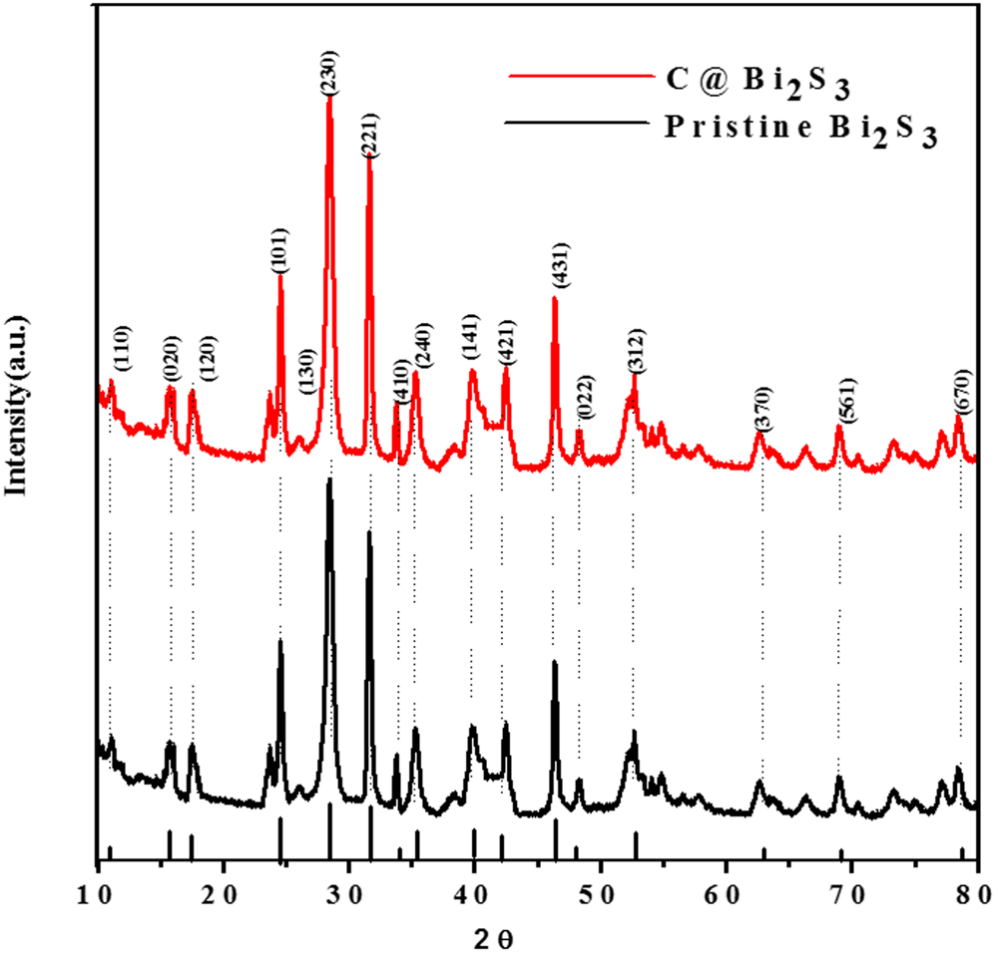
XRD pattern of pristine Bi_2_S_3_ and C@Bi_2_S_3_ samples at 8 h.

The phase purity and structural features of the as-synthesized samples were verified via XRD, and the results are shown in Fig. 6. All the diffraction peaks in the both XRD patterns can be indexed to the orthorhombic phase of Bi_2_S_3_, which was well matched with JCPDS No. 89–8964. Interestingly, the weak peaks also match well with the standard pattern. In case of the C@Bi_2_S_3_ sample, similar diffraction peaks were observed owing to the amorphous nature of the carbon materials. As observed in the XRD result, the intensities of the diffraction peaks at 2θ = 11.08, 15.72, 17.56, 23.71, 24.92, 28.64, 31.11, 33.92, 35.91, 39.89, 42.69, 46.5, 48.44, 52.76, 62.71, 69.44, and 78.46 degrees corresponding to the (110), (020), (120), (101), (130), (230), (221), (410), (240), (141), (421), (431), (022), (312), (370), (561), and (670) planes of orthorhombic Bi_2_S_3_ respectively, agree well with reported values in literature^28^. Both the pristine Bi_2_S_3_ and C@Bi_2_S_3_ samples exhibit good crystallinity and all the peaks can be clearly attributed to the orthorhombic phase of Bi_2_S_3_. Carbon is not recognized in the pattern of the C@Bi_2_S_3_ sample, demonstrating the disordered nature of the carbon layer^31^. In fact, the carbon component in the as-prepared C@Bi_2_S_3_ sample is confirmed via SEM and HRTEM elemental mapping.

**Figure 7:**
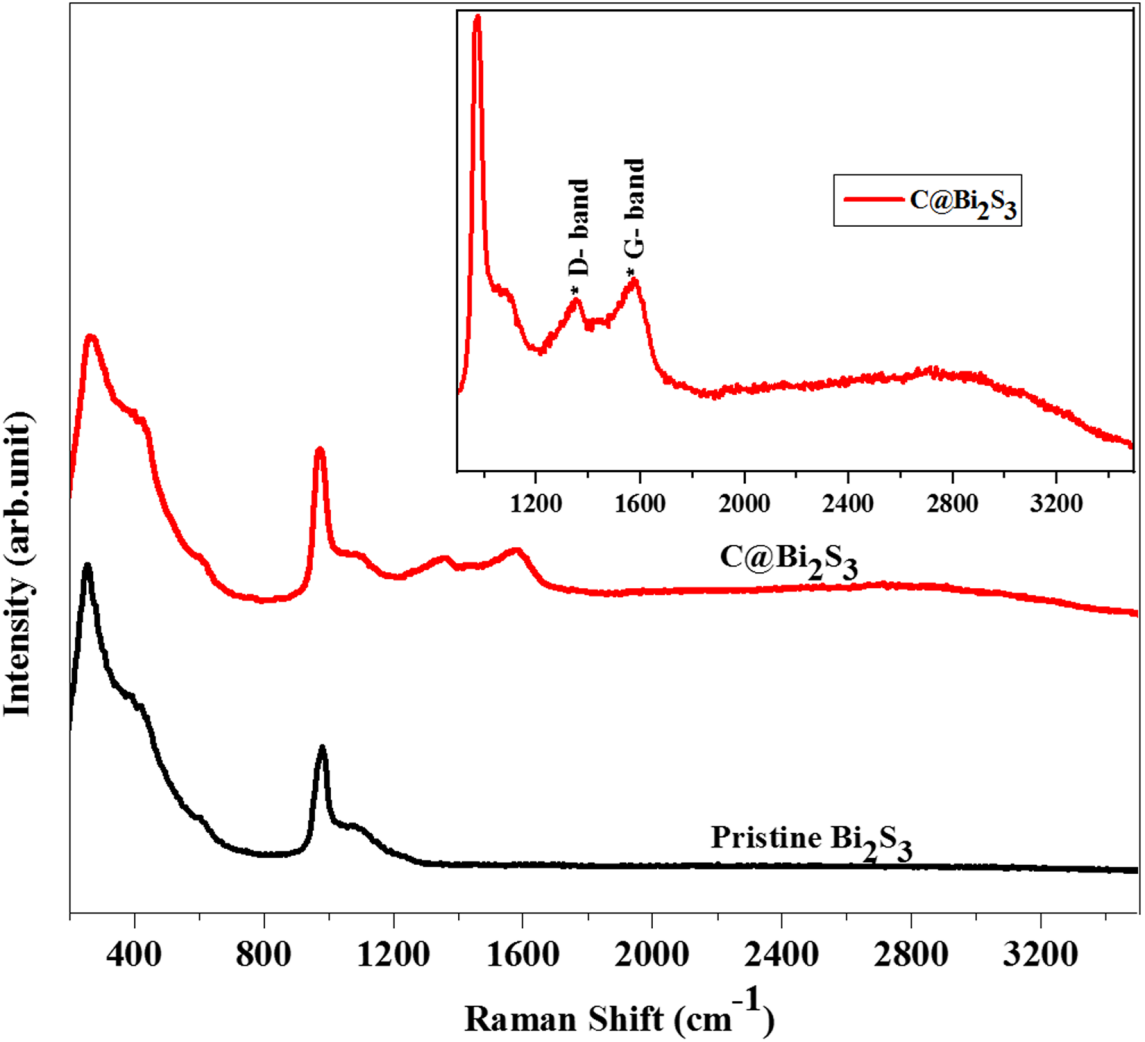
Raman spectra of pristine Bi_2_S_3_ and C@Bi_2_S_3_ samples at 8 h.

To confirm the nature of carbon shell existing on the Bi_2_S_3_ nanorods, Raman spectroscopy of pristine Bi_2_S_3_ and C@Bi_2_S_3_ samples was recorded. As shown in Fig. 7, both samples demonstrate scattering bands at 258, 347, 426, 632, and 963 cm^−1^, which are well consistent with the reported literatures for the crystalline Bi_2_S_3_^32^. From the partial enlarged drawing of C@Bi_2_S_3_ Raman spectra (inset of Fig. 7), two weak peaks appeared at 1354 and 1575 cm^−1^ are observed. These two new peaks of C@Bi_2_S_3_ sample are ascribed to the D and G bands of carbon, respectively^13^. Thus the Raman confirms the presence of carbon in the synthesized C@Bi_2_S_3_ sample.

**Figure 8:**
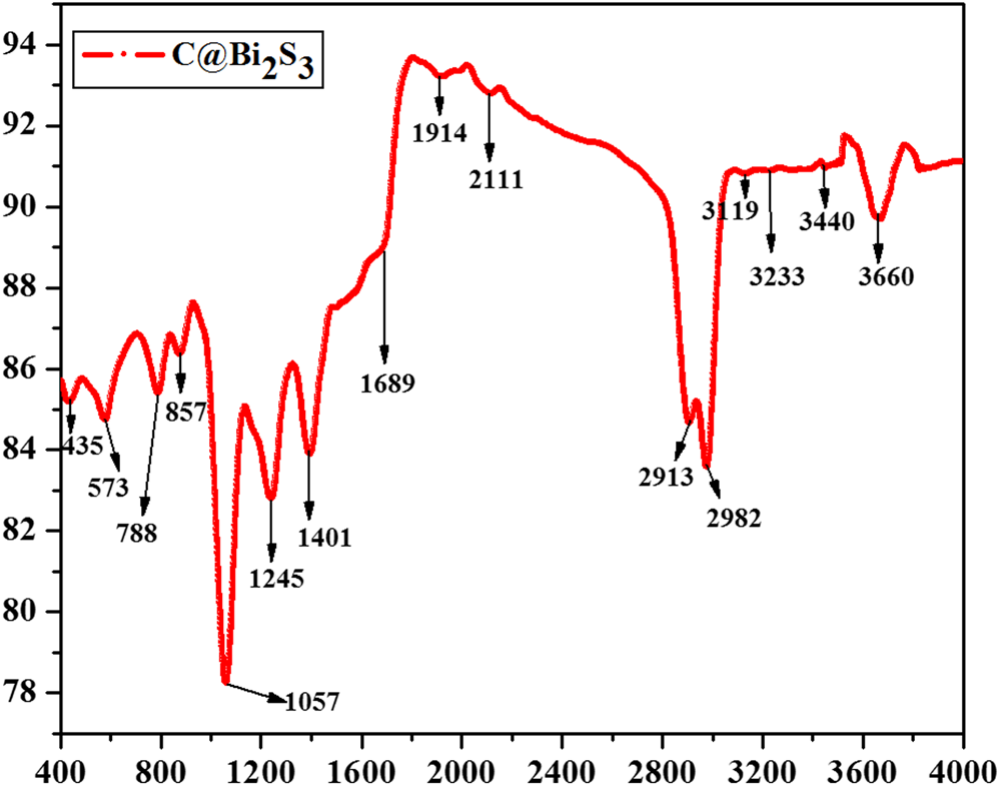
FTIR spectrum of the C@Bi_2_S_3_ sample at 8 h.

The FTIR spectrum of the C@Bi_2_S_3_ core-shell structure within the range 400–4000 cm^−1^ is shown in Fig. 8. The peaks at 435, 573, 788, and 857 cm^−1^ can be assigned to Bi-S bonding^28^. The vibrational stretching peak at 1045–1250 cm^−1^ can be attributed to the asymmetric S-H bond of Bi_2_S_3_. As reported in the previous study^33^, the existence of polymerized carbon layer could be identified through the FTIR analysis (Fig. 8). The carbon layer is identified by the appearance of C=O, C=C, O-H, and C-OH modes of vibration peaks at 1689, 1914, 3110–3670, and 1410 cm^−1^, respectively. This indicates the presence of a large number of residual hydroxyl groups and intermolecular hydrogen bonds, which reflects the dispersion of water molecules.

**Figure 9:**
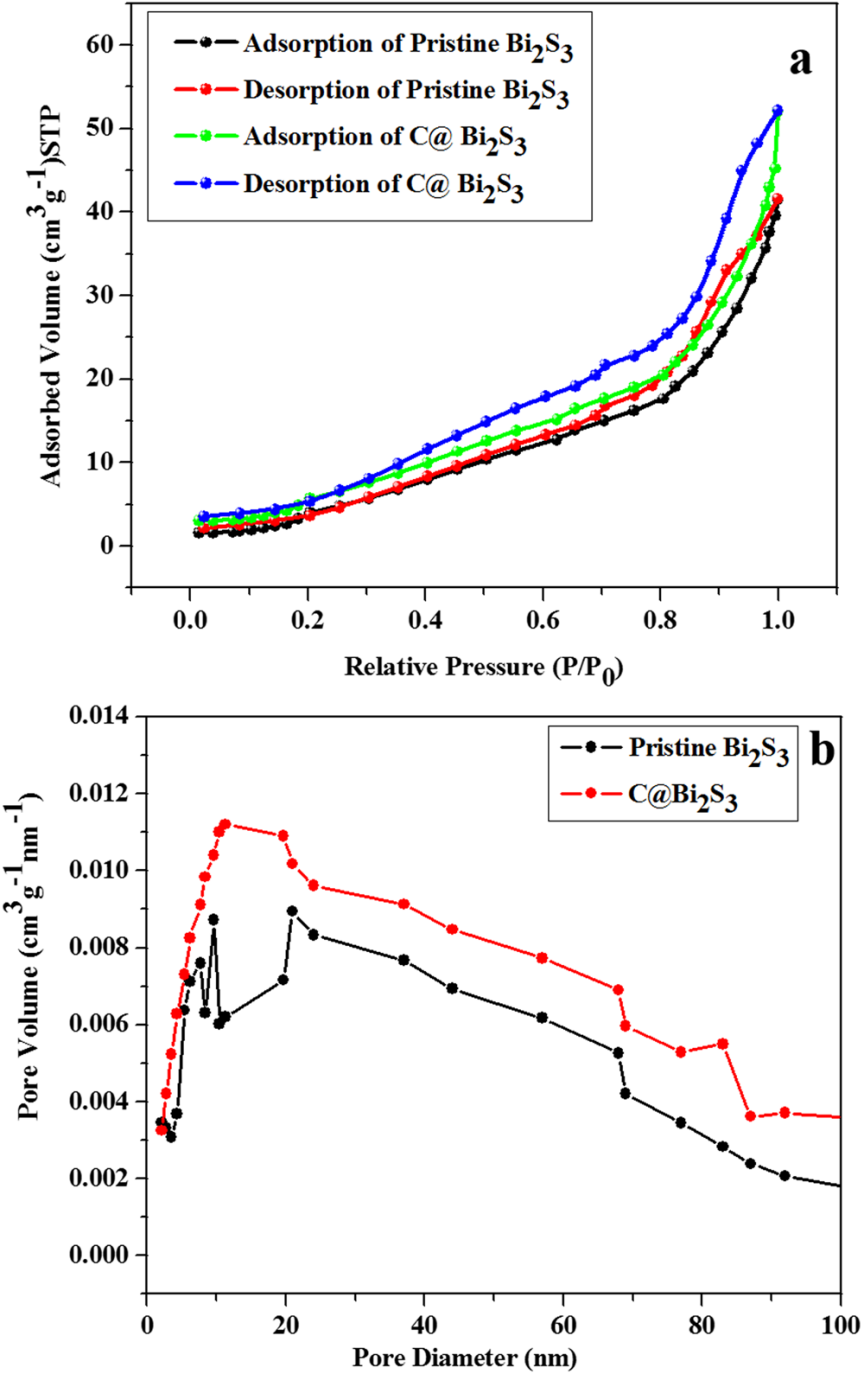
N_2_ adsorption/desorption isotherm and, (**b**) Barrett–Joyner–Halenda (BJH) pore size distribution data of the pristine Bi_2_S_3_ and C@Bi_2_S_3_ samples at 8 h.

The textural features of pristine Bi_2_S_3_ and C@Bi_2_S_3_ were investigated using N_2_ adsorption-desorption isotherms present in type-H3 hysteresis loops (Fig. 9a), which indicates the typical mesoporous characteristics. The Brunauer−Emmett−Teller (BET) surface areas of the pristine Bi_2_S_3_ and C@Bi_2_S_3_ samples estimated from N_2_ isotherms were determined to be 22.58 and 43.63 m^2 ^g^−1^, respectively. Compared to the previously reported Bi_2_S_3_@C (surface area is 11.2 m^2^ g^−1^), the asprepared C@Bi_2_S_3_ possess higher BET surface area^13^. The Barrett−Joyner−Halenda (BJH) pore-size distribution curves (Fig. 9b) of the pristine Bi_2_S_3_ and C@Bi_2_S_3_ samples display an average pore size of 15.1 and 16.2 nm, respectively, which further reveals their mesoporous features. The enhancement of the specific surface area and pore size by the introduction of carbon material characteristics clearly indicates that the C@Bi_2_S_3_ sample can store more charge for enhanced specific capacity owing to the enlarged active sites and the good approachability of electrolyte ions.

**Figure 10:**
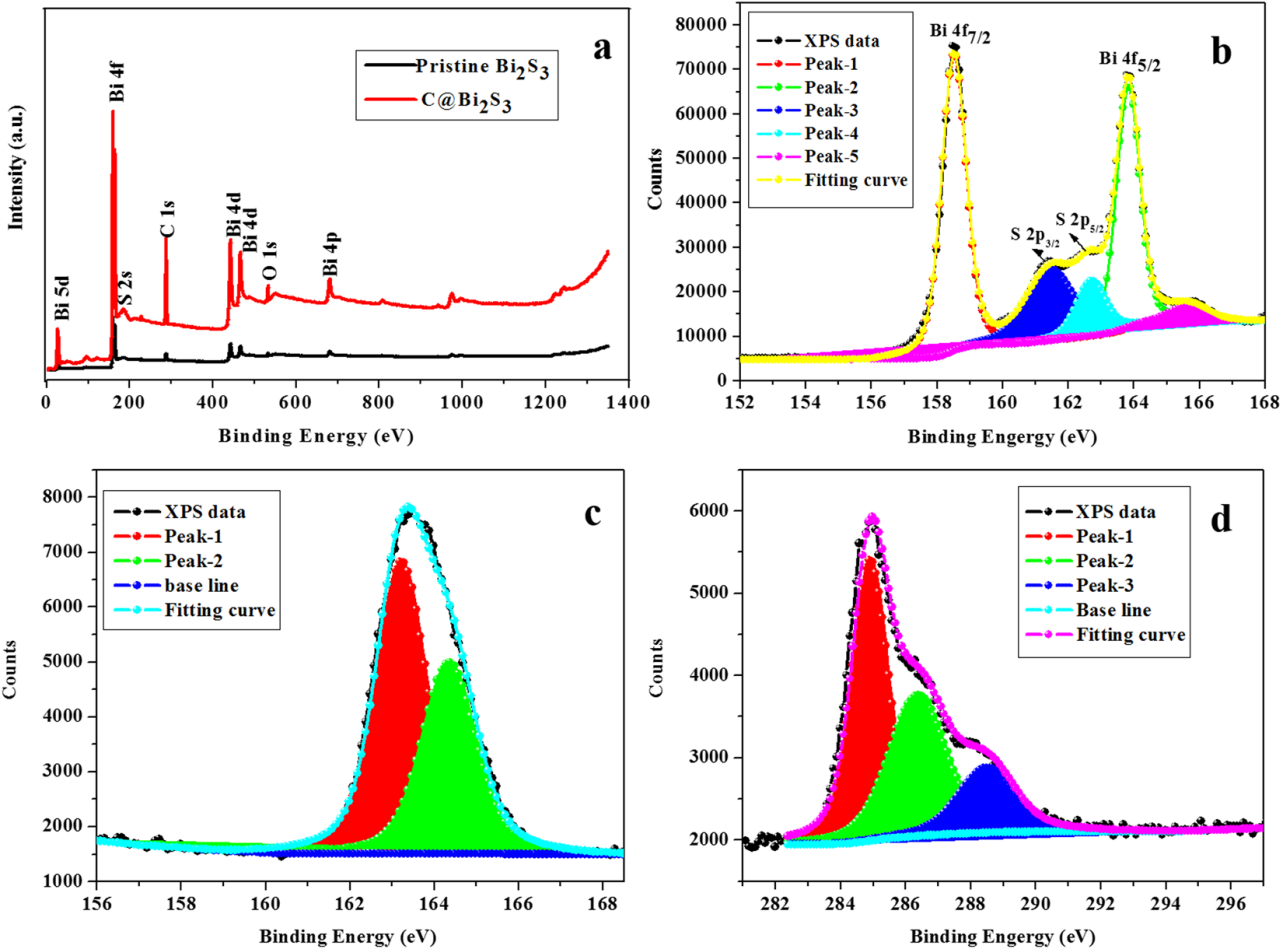
XPS survey spectra of the pristine Bi_2_S_3_ and C@Bi_2_S_3_ samples (**a**) and high-resolution scan of Bi 4f (**b**), S 2p (**c**), and C 1s (**d**) of the C@Bi_2_S_3_ sample at 8 h.

The chemical composition and chemical status of the elements in synthesized C@Bi_2_S_3_ core-shell structure were measured using x-ray photoelectron spectroscopy, as shown in Fig. 10. The Fig. 10a displays the XPS survey spectra of the pristine Bi_2_S_3_ and C@Bi_2_S_3_ samples. The prominent peaks at 158.56 and 163.81 eV in Fig. 10b are typical for Bi 4f_7/2_ and Bi 4f_5/2_, respectively, while the peaks at 161.34 and 162.52 eV are ascribed to the divalent anionic state of sulfur in Bi_2_S_3_. Figure 10c presents the peak at 163.53 eV corresponding to the binding energies of S 2p. The C 1s scan for carbon displayed in Fig. 10d has peaks at 284.53, 286.08, and 288.54 eV. The C 1s peak at 284.53 eV is dominated by elemental carbon, while two shoulders located at 286.08 and 288.54 eV are ascribed to the existence of C–OH and C–O–C bonds, respectively^33^. The presence of these small peaks suggests the existence of some residual groups due to the insufficient reduction of D-glucose^34,35^.

**Figure 11:**
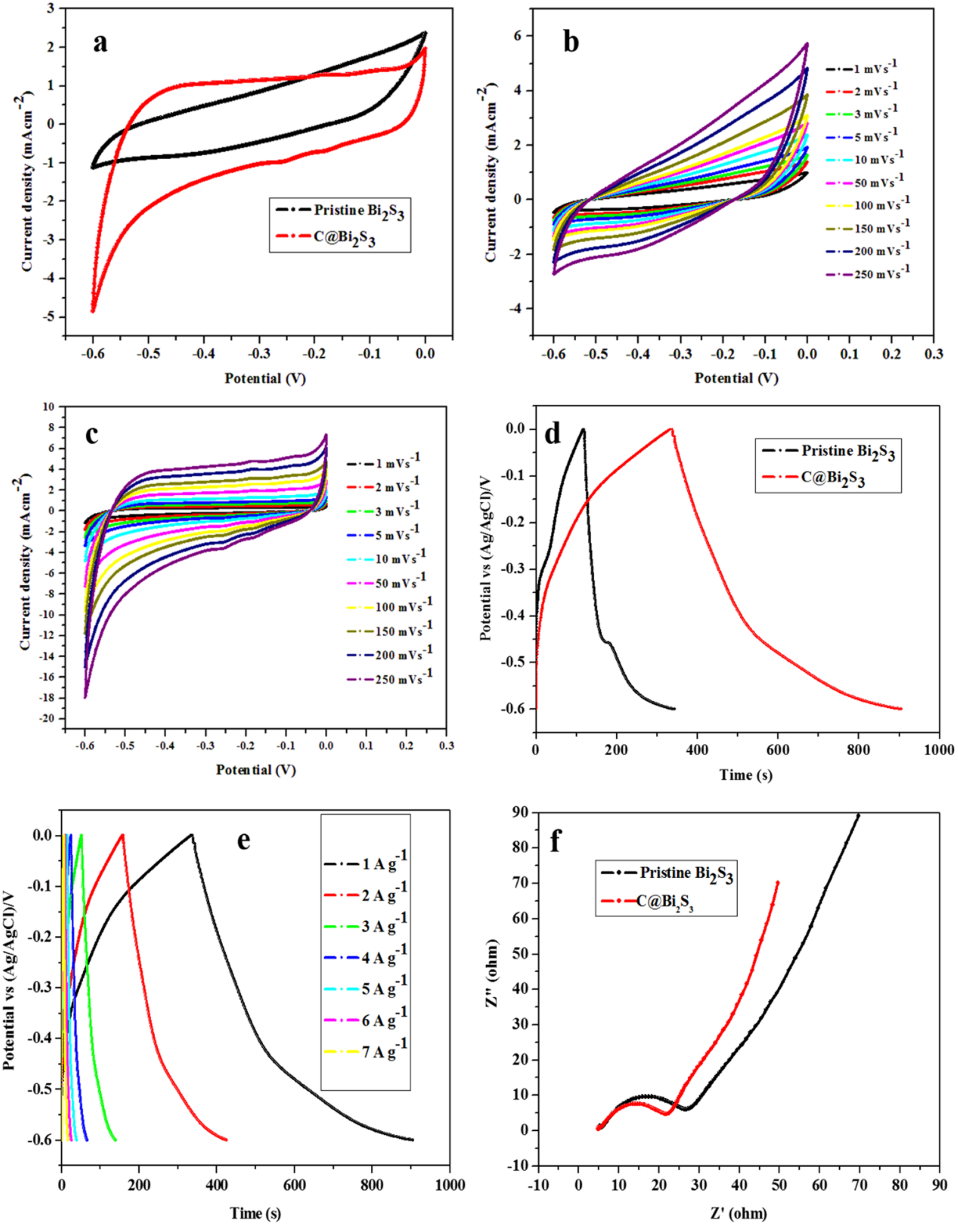
(**a**) CV curves at 10 mV s^−1^, (**b,c**) CV curves at different scan rates, and (**d**) charge-discharge curves at 4 A g^−1^ of the pristine Bi_2_S_3_ and C@Bi_2_S_3_ samples at 8 h; (**e**) charge-discharge curves of the C@Bi_2_S_3_ sample at 8 h at different current densities, and (**f**) EIS spectra of pristine Bi_2_S_3_ and C@Bi_2_S_3_ samples at 8 h.

In order to estimate the electrochemical properties of the pristine Bi_2_S_3_ and C@Bi_2_S_3_ samples, cyclic voltammetry (CV) and galvanostatic charge/discharge tests, electron impedance, and photocurrent tests were performed. Figure 11a presents the CV curves for the pristine Bi_2_S_3_ and C@Bi_2_S_3_ core-shell structures at a scanning rate of 10 mV s^−1^ for a potential ranging from −0.6 to 0 V (vs SCE) in a 0.5 M Na_2_SO_4_ electrolyte. As shown in Fig. 11a, the area of the C@Bi_2_S_3_ core-shell structure is larger than that of pristine Bi_2_S_3_, representing a greater specific capacitance. Figure 11(b,c) displays the CV curves of the pristine Bi_2_S_3_ and C@Bi_2_S_3_ core-shell structures at various scanning rates. The voltammograms were recorded between −0.6 and 0 V at potential scan rates ranging from 1 to 250 mV s^−1^ in a 0.5 M Na_2_SO_4_ solution. No redox peak is observed at different scan rates in the CV curves, suggesting that the electrode is charged and discharged at a pseudo-constant rate over the complete voltammetric cycle. The CV curve at faster scan rates has a larger area with a greater charge capacitance compared with that at a lower scan rate.

Figure 11d presents the constant current charge/discharge curves of the pristine Bi_2_S_3_ and C@Bi_2_S_3_ samples at a current density of 1 A g^−1^. During the charging and discharging steps, the charge curve of the samples were not symmetric to its corresponding discharge counterpart with a negligible internal resistance (IR) drop, indicating the supercapacitive phenomenon. The GCD curve of the C@Bi_2_S_3_ sample exhibits a longer discharging time compared with the charging time. This phenomenon is repeatedly observed in Bi_2_S_3_-based electrode systems^36^; however, the investigation of the exact mechanism of this phenomenon is still under progress. In addition, the GCD curves of the C@Bi_2_S_3_ sample at different current densities (1, 2, 3, 4, 5, 6 and 7 A g^−1^) is presented in Fig. 11e.

The EIS analysis has been recognized as one of the principal methods to investigate the fundamental behavior of electrode materials, especially for supercapacitors. The impedance of both the samples are tested at frequencies varying between 0.1 Hz and 1 MHz at an open circuit potential with an AC perturbation of 0 mV. Figure 11f shows the Nyquist plots of the pristine Bi_2_S_3_ and C@Bi_2_S_3_ electrodes. Both the samples demonstrated a semicircular arc at higher frequencies and a straight line at lower frequencies, indicating that the electrode processes are organized by the charge transfer (i.e., electrochemical reaction) in the former region and by the diffusion of charges (i.e., mass transfer) in the absolute region. The intercept on the Z’ axis refers to R_s_, including the solution resistance and the contact resistance, whereas the dimension of the semicircle reflects the electrode film resistance (R_f_) and charge transfer resistance (R_ct_), which are consistent with reported literature^13^. EIS parameters, including the solution resistance (R_s_), charge transfer resistance (R_ct_), and time constant (τ) were obtained as 1.2 Ω, 5.02 Ω, and 0.32 ms, respectively, for the C@Bi_2_S_3_ electrode, indicating fast charge/discharge processes, small solution resistance, and superior electrical conductivity at the electrode/electrolyte interface. Therefore, the carbon layer can efficiently bind with Bi_2_S_3_ nanorods, and thus improve electrical contact with the current collector^13^. This confirms that more active carbon participates in the charge/discharge reaction process, leading to a higher charge carrier mobility.

**Figure 12:**
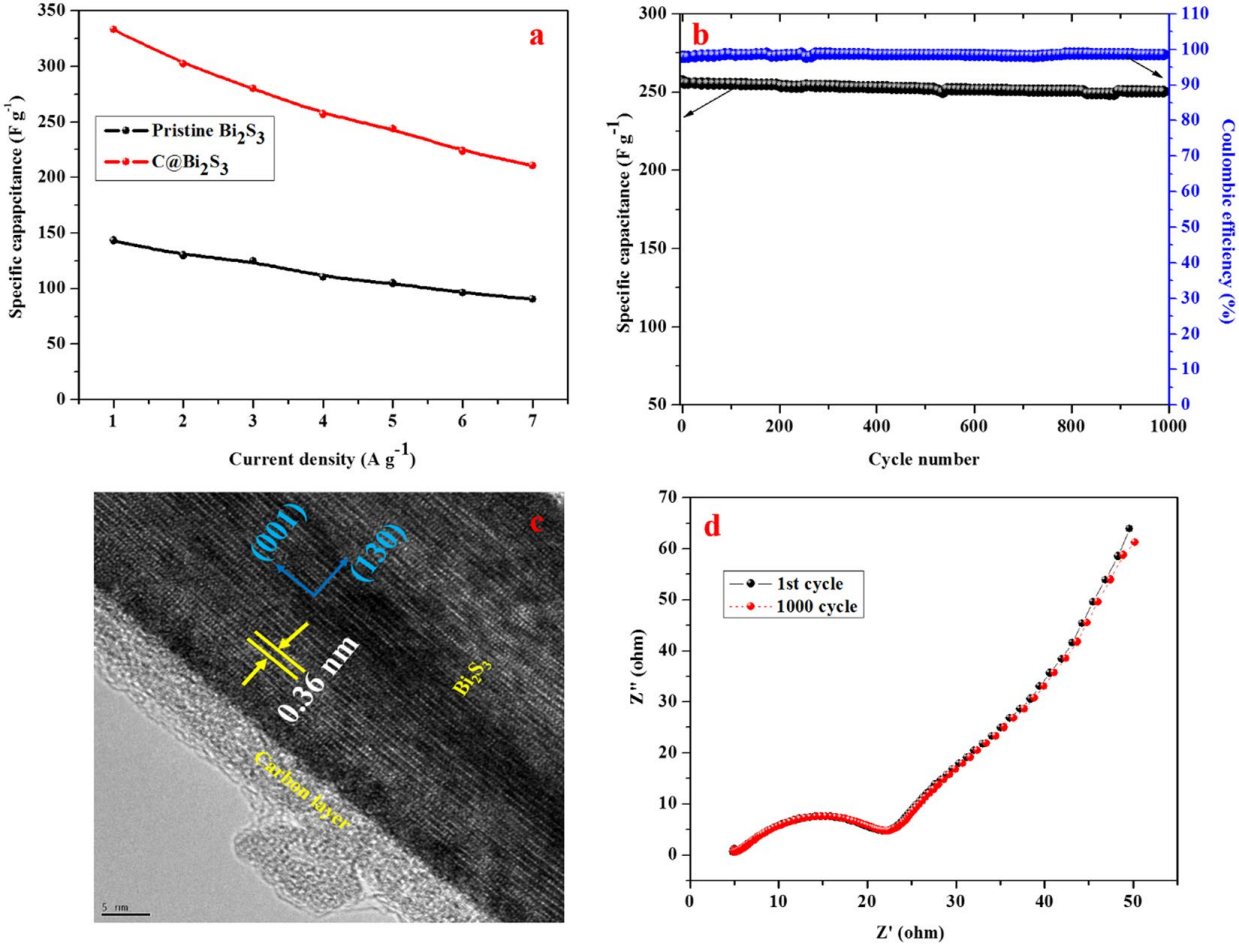
(**a**) The calculated specific capacitance of the pristine Bi_2_S_3_ and C@Bi_2_S_3_ electrodes at 8 h as a function of current densities, (**b**) specific capacitance retention and coulombic efficiency of the C@Bi_2_S_3_ electrodes at 8 h, (**c**) HRTEM image and (**d**) EIS spectra of the C@Bi_2_S_3_ electrodes at 8 h after 1000 cycles.

Figure 12a presents the relationship between the capacitance and current density. The specific capacitance is 143.24, 129.87, 124.69, 110.45, 104.83, 96.21, and 90.61 F g^−1 ^for pristine Bi_2_S_3_ electrode, while the specific capacitance of the C@Bi_2_S_3_ electrode is 333.43, 302.21, 280.16, 257.03, 243.97, 223.89, and 210.84 F g^−1^ at 1, 2, 3, 4, 5, 6, and 7 A g^−1^, respectively. The obtained value of the specific capacitance of the C@Bi_2_S_3_ electrode is higher than the previously reported values for carbon-decorated MoS_2_ (210 F g^−1^ at 1 A g^−1^)^37^. As the current density increased from 1 to 7 A g^−1^, the specific capacitance linearly decreased to 90.6 and 210.84 F g^−1^ with a 63.2% retention of its original value. These findings specify that both the electrodes have a remarkable rate capability.

To explore the electrochemical stability and durability of the C@Bi_2_S_3_ core-shell structure, consecutive GCD cycles were conducted at a constant current density of 4 A g^−1^. Figure 12b presents the relationship between the specific capacitance and coulombic efficiency with respect to the number of cycles. The specific capacitance remained 97.36% of initial value even after 1000 cycles of operation. In addition, the morphology of C@Bi_2_S_3_ core-shell structure was examined using HRTEM, as shown in Fig. 12c. It can be seen that the core-shell feature of C@Bi_2_S_3_ still maintained even after suffering intense cycling test, which contributes to the remarkable cyclic stability. Moreover, the EIS of the 1^st^ and 1000^th^ cycle samples was conducted and compared. Figure 12d shows the Nyquist plots for the C@Bi_2_S_3_ electrode before and after 1000 cycles. It can be seen that both the curves contained a vertical line parallel to the imaginary axis at a low frequency, signifying a pseudocapacitor behavior^38,39^. In addition, the R_ct_ value was consistent before and after 1000 cycles (5.02 and 5.08 Ω), respectively, which is reliable with the remarkable stability, as confirmed by the cycling tests, suggesting the interconnected structure of core-shell features of C@Bi_2_S_3_.

**Figure 13:**
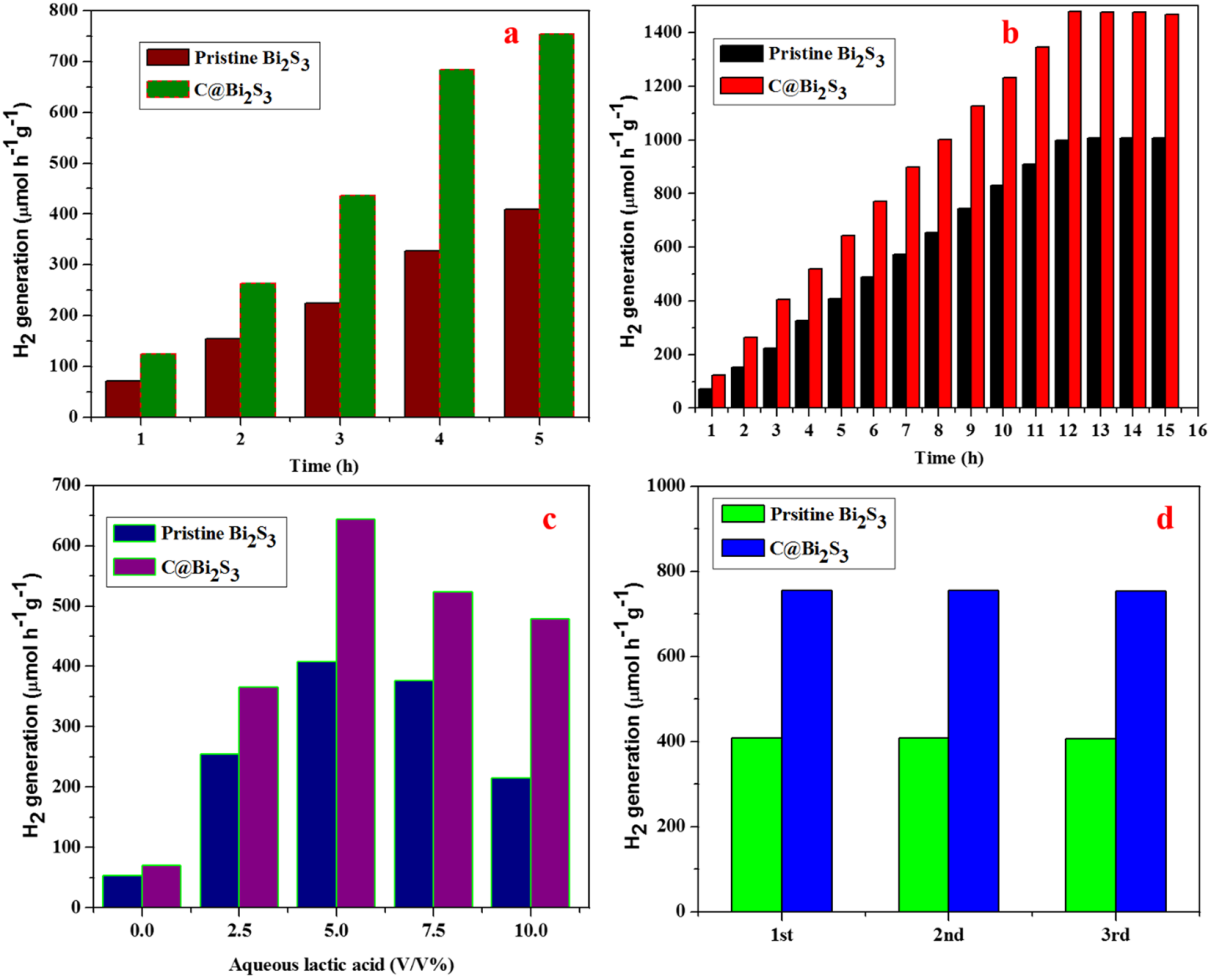
(**a**) Photocatalytic H_2_ production and, (**b**) Time on stream photocatalytic H_2_ production activity over the pristine Bi_2_S_3_ and C@ Bi_2_S_3_ samples under simulated solar light irradiation in 5 vol% lactic acid: water solution, (**c**) effect of lactic acid concentration on the photocatalytic H_2_ production over 5 mg of pristine Bi_2_S_3_ and C@ Bi_2_S_3_ samples and (**d**) recycling studies over the pristine Bi_2_S_3_ and C@Bi_2_S_3_ samples under simulated solar light irradiation in 5 vol% lactic acid.

Figure 13a presents the photocatalytic H_2_ production over the pristine Bi_2_S_3_ and C@Bi_2_S_3_ samples via water splitting using a 5 vol% lactic acid solution as a sacrificial agent. Due to the low bandgap, the recombination of electrons and holes in the Bi_2_S_3_ semiconductor is rapid leading to lower quantum yields. Here the carbon shell on the Bi_2_S_3_ prevents the recombination process by accepting an electron from the conduction band of Bi_2_S_3_. Also the high conductivity of carbon shell affluences the transportation of electrons to the reaction center that is the surface of carbon shell where the proton reduction process occurs. It is obvious that the photocatalytic H_2_ generation increases with an increase in the irradiation time. Interestingly, the amount of H_2_ generation is higher over the core-shell structure of C@Bi_2_S_3_ (754.34 µmol h^−1^ g^−1^) compared to the pristine Bi_2_S_3_ (408.54 µmol h^−1^ g^−1^), which is a 1.84-fold improvement in H_2_ production over the core-shell structure of C@Bi_2_S_3_. It is believed that the improved photocatalytic activity may be associated with the core-shell feature of C@Bi_2_S_3_, which leads to close contact between the organic/inorganic frame networks. This core-shell interconnected assembled structure could also transport photo-induced electrons and holes to the binding sites; thus, the efficient oxidation of water occurred in the presence of lactic acid as a sacrificial agent, which is also consistent with previously reported literatures^40–42^. In addition, the core-shell heterostructure may facilitate remarkable electron transfer compared with the pristine one, which can efficiently improve hydrogen production^43^. As it already discussed in Fig. 10f in which the EIS of the C@Bi_2_S_3_ sample showed lower semicircle as compared to the pristine Bi_2_S_3_. The lower arc for the C@Bi_2_S_3_ sample is resulted from the less resistance of the material for the charge transfer process which is crucial for the electron transfer to the protons during the water splitting reaction to produce hydrogen. Thus, the presence of carbon shell improves the charge transfer characteristics of Bi_2_S_3_ that might be the reason for enhanced activity.

The effect of carbon shell thickness on the photocatalytic activity for the hydrogen production has been studied over C@Bi_2_S_3_ catalysts prepared at various reaction times (Fig. S1). All the C@Bi_2_S_3_ photocatalysts showed superior H_2_ production than the pristine Bi_2_S_3_. This infers that the presence of carbon shell has strong influence on the enhanced photocatalytic activity of Bi_2_S_3_. Maximum H_2_ production was observed over the C@Bi_2_S_3_–8h catalyst which was prepared at 8 h of reaction time. However, the C@Bi_2_S_3_−12h sample prepared at 12 h reaction time showed slightly lower photocatalytic activity than the C@Bi_2_S_3_−8h sample. The excess reaction time might have resulted in the formation of overgrown thick carbon shell over the Bi_2_S_3_ in which the photoexcited electron in the Bi_2_S_3_ core has to travel longer path to reach the surface of the carbon shell where the proton reduction takes place. This results in the recombination of excited electrons with the holes and subsequent reduction in the photocatalytic activity. Also the excess reaction time might have formed individual carbon particles which may not be in direct contact with the C@Bi_2_S_3_ core shell structure. In the present study, the C@Bi_2_S_3_ core shell material prepared at 8 h reaction time has shown the optimum photocatalytic activity and hence we believe that 8 h reaction time is ideal for the growth of carbon shell on the surface of Bi_2_S_3_ core. Thus the C@Bi_2_S_3_−8h catalyst was selected for further studies.

Time on stream activity over the pristine Bi_2_S_3_ and C@Bi_2_S_3_ catalysts are conducted as shown in the Fig. 13b. It is clearly seen from the data that the activity is found to increase linearly up to 10 h and then tends to stable after 10 h of reaction. The reduction in the rate of hydrogen production with the time may be seen due to the pressure advanced by the produced hydrogen during reaction. However, when the produced gases were evacuated and purged with N_2_ for every five hours the activity of the catalyst is retained. In order to optimize the lactic acid concentration, reactions were carried out over pristine Bi_2_S_3_ and C@Bi_2_S_3_ catalysts using lactic acid: water mixtures with different lactic acid amounts. The results are summarized in Fig. 13c. It displays that the lactic acid concentration is affecting the production of hydrogen and lactic acid is involved in the production of H_2_. The photocatalytic hydrogen rate increases with the increase of initial lactic acid concentration. The optimal concentration of lactic acid is at 5% with a photocatalytic hydrogen rate of 408.54 and 754.34 µmol h^−1^ g^−1^ over the pristine Bi_2_S_3_ and C@Bi_2_S_3_ catalysts, respectively. And with increase in lactic acid concentration over 5%, no increase is seen in H_2_ production. The reason may be that at higher concentrations the surface of the photocatalyst extents saturation with no further improve in H_2_ production.

For practical applications, the reusability and stability of as-prepared photocatalysts is a key parameter. Figure 13d presents the recycling of both photocatalyst samples up to three successive cycles. The results of this recycling test indicates that after the third cycle, no significant decline was observed in the hydrogen generation rate, which reflects that the as-prepared samples are more stable, including those used in cyclic operations. Therefore, it can be concluded that the as-prepared samples, especially, C@Bi_2_S_3_ core-shell structures are more stable and reusable visible light–driven photocatalysts, which represent a fruitful methodology for harvesting solar energy for H_2_ evolution and supercapacitor applications.

**Figure 14:**
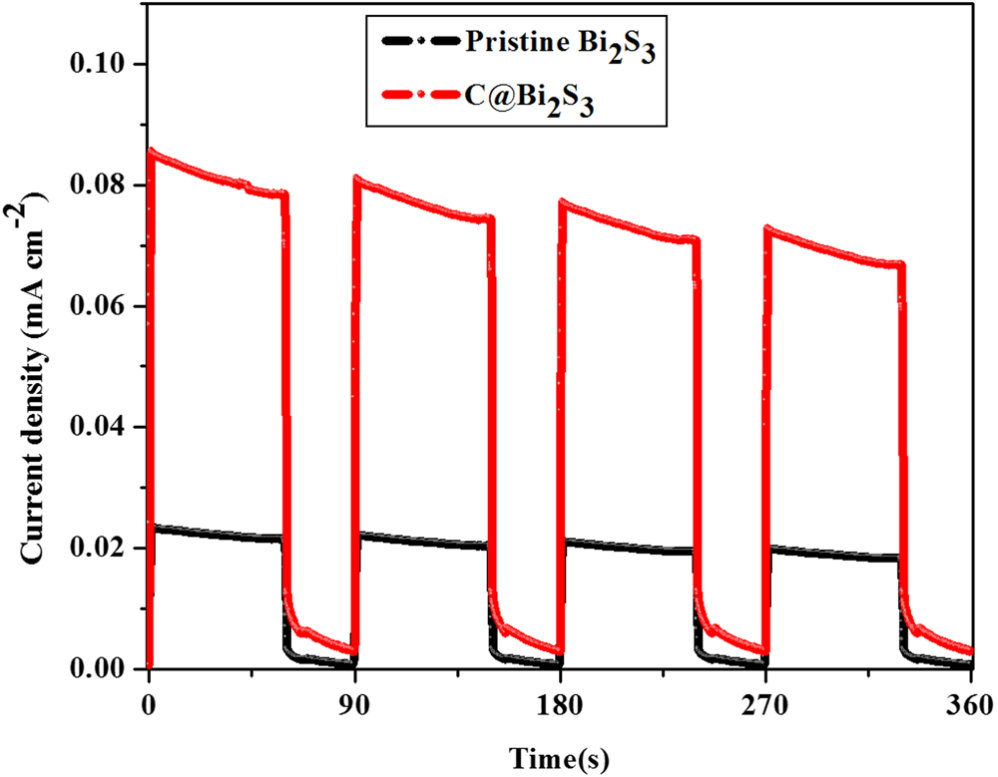
Transient photocurrent studies of the pristine Bi_2_S_3_ and C@Bi_2_S_3_ samples.

For a deeper insight into the behavior of photogenerated charge carriers, the profiles of transient photocurrent vs. time were obtained for the as-prepared Bi_2_S_3_ and C@Bi_2_S_3_ samples. The charge carrier separation phenomena of these samples were tested via on/off photocurrent at a bias voltage of 0.4 mV under simulated solar light irradiation, as shown in Fig. 14a. The results indicate that the C@Bi_2_S_3_ core-shell structure exhibits a remarkably higher photocurrent compared to the pristine Bi_2_S_3_, which is consistent with the above electrochemical performance (Fig. 11). The higher photocurrent over the core-shell structure of C@Bi_2_S_3_ specified that there was effective transfer of interfacial photogenerated charge carriers from Bi_2_S_3_ and improvement in electron-hole separation^18^. This time-dependent response of the C@Bi_2_S_3_ sample is preferable for optoelectronic device applications. The photoexcited electron and holes can facilitate free radicals such as superoxide radicals and holes, which have been previously confirmed by electrochemical performance tests (Fig. 11). All these results confirm that more electrons may participate for photocatalytic water splitting and H_2_ generation.

**Figure 15:**
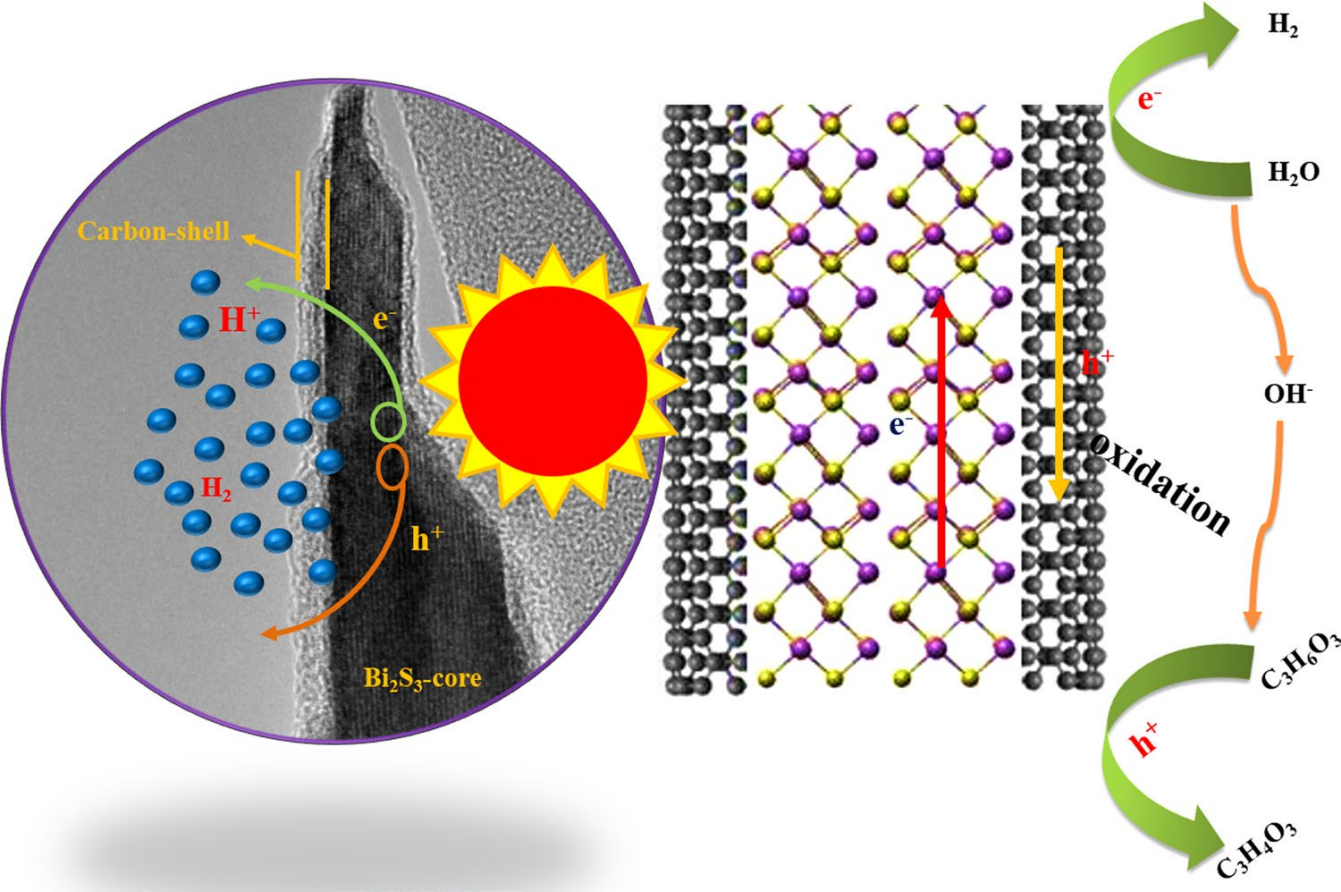
Schematic illustration of the plausible mechanism of photocatalytic H_2_ production over the C@Bi_2_S_3_ core-shell structure under simulated solar light irradiation.

Based on the above results and discussion, a plausible mechanism of photocatalytic performance can be described as shown in Fig. 15. When C@Bi_2_S_3_ is exposed to visible light irradiation, Bi_2_S_3_ is excited and generates electron–hole pairs. The holes generated on the Bi_2_S_3_ nanorods can easily transfer to carbon because of the intimate contact between carbon and Bi_2_S_3_. Meanwhile, carbon can serve as a catalytic site for H_2_ evolution. The following reasons can clarify the improved photocatalytic hydrogen evolution rate for the C@Bi_2_S_3_: (i) improved visible light absorption property helps in producing more electrons and holes, which advances photocatalytic activity, (ii) the remarkable specific surface area of the C@Bi_2_S_3_ core-shell structure provide more reactive sites for promoting hydrogen evolution, (iii) skillfully designed heterostructures prominently endorse the separation of electrons and holes, and (iv) the excellent properties of carbon materials such as high conductivity and more active sites benefit charge carrier separation and charge transfer to diminish the electron–hole pair recombination and enhance the photocatalytic hydrogen evolution rate. Finally, the introduction of carbon can reduce the H^+^ reduction overpotential and act as a catalytic active site.

## Conclusions

We attempted and demonstrated a promising core-shell design of C@Bi_2_S_3_ nanorods. The as-fabricated core-shell heterostructure can be directly employed as an electrode for supercapacitors, demonstrating a remarkable specific capacity of 333.43 F g^−1^ at a current density of 1 A g^−1^ and a considerable cycling stability with a capacity retention of 97.36% after 1000 cycles at a current density of 4 A g^−1^. The remarkable cycling performance, combined with robust rate capability, exhibit the potential of C@Bi_2_S_3_ to be used as a novel electrode material for a supercapacitor. In addition, the core-shell structure of the C@Bi_2_S_3_ electrode demonstrated a H_2_ evolution rate of 754.34 µmol h^−1^ g^−1^, a 1.84-fold improvement over the pristine Bi_2_S_3_. This outstanding improvement was due to enhanced specific surface area with site edges of carbon that helps in the effective separation and transportation of photoinduced carriers. It is expected that the proposed synthetic strategy has potential for the exploration of advanced electrochemical cell materials.

## Electronic supplementary material

Supplementary Information
