# Supplementary material for: Sacrificial-template-free synthesis of core-shell C@Bi2S3 heterostructures for efficient supercapacitor and H2 production applications
Source: Sci Rep. 2018 Mar 8;8:4194. doi: 10.1038/s41598-018-22622-0 (PMC5843642; doi:10.1038/s41598-018-22622-0)
Supplement: Supplementary file 1 — Supplementary Information [file 41598_2018_22622_MOESM1_ESM.docx]

**Supplementary Information**

**Sacrificial**-**template**-**free synthesis of core**-**shell C@Bi_2_S_3_ heterostructures for efficient supercapacitor and H_2_ production applications**

S.V. Prabhakar Vattikuti^1^*, Police Anil Kumar Reddy^2^, Jaesool Shim^1^* and Chan Byon^2^*

*^1^School of Mechanical Engineering, Yeungnam University,* *Gyeongsan 712-749, South Korea*

*^2^School of Mechanical and Nuclear Engineering, Ulsan National Institute of Science and Technology (UNIST),* *Ulsan 44919, Republic of Korea*

***Corresponding author Address:**

Dr. S.V. Prabhakar Vattikuti,

School of Mechanical Engineering

Yeungnam University 214-1 Dae-dong Gyeongsan-si,

Gyeongsangbuk-do (712-749, Republic of Korea)

Mobile: +82-(0)53-810-2452

Fax: +82-53-810-4627

*Corresponding author E-mail: [drprabu@ynu.ac.kr](mailto:drprabu@ynu.ac.kr) (S.V. Prabhakar Vattikuti), [jshim@ynu.ac.kr](mailto:jshim@ynu.ac.kr) (Jaesool Shim) and [cbyon@unist.ac.kr](mailto:cbyon@unist.ac.kr) (Chan Byon)





**Fig. S1.** Photocatalytic activity for H_2_ production over Pristine Bi_2_S_3_ and different C@Bi_2_S_3_ catalysts prepared at various reaction times.
